# Graphite: painting genomes using a colored de Bruijn graph

**DOI:** 10.1093/nargab/lqae142

**Published:** 2024-10-23

**Authors:** Rick Beeloo, Aldert L Zomer, Sebastian Deorowicz, Bas E Dutilh

**Affiliations:** Theoretical Biology and Bioinformatics, Utrecht University, Padualaan 8, 3584 CH Utrecht, The Netherlands; Department of Infectious Diseases and Immunology, Faculty of Veterinary Medicine, Utrecht University, 3584 Utrecht, The Netherlands; Department of Algorithmics and Software, Silesian University of Technology, Akademicka 16, Gliwice PL-44100, Poland; Theoretical Biology and Bioinformatics, Utrecht University, Padualaan 8, 3584 CH Utrecht, The Netherlands; Institute of Biodiversity, Faculty of Biological Sciences, Cluster of Excellence Balance of the Microverse, Friedrich Schiller University Jena, 07743 Jena, Germany

## Abstract

The recent growth of microbial sequence data allows comparisons at unprecedented scales, enabling the tracking of strains, mobile genetic elements, or genes. Querying a genome against a large reference database can easily yield thousands of matches that are tedious to interpret and pose computational challenges. We developed Graphite that uses a colored de Bruijn graph (cDBG) to paint query genomes, selecting the local best matches along the full query length. By focusing on the best genomic match of each query region, Graphite reduces the number of matches while providing the most promising leads for sequence tracking or genomic forensics. When applied to hundreds of *Campylobacter* genomes we found extensive gene sharing, including a previously undetected *C. coli* plasmid that matched a *C. jejuni* chromosome. Together, genome painting using cDBGs as enabled by Graphite, can reveal new biological phenomena by mitigating computational hurdles.

## Graphical Abstract

**Graphical Abstract:**
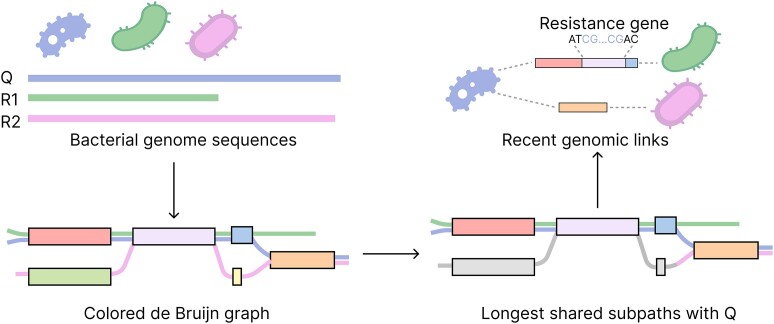

## Introduction

Tracking genetic sequences is crucial for understanding various biological phenomena, such as recombination and horizontal gene transfer (HGT) of genetic elements, as well as source attribution in epidemiology. Advances in DNA sequencing, coupled with the abundance of sequencing data in databases, enable us to reconstruct the movement of genetic information at a range of scales. For example, the rearrangement of chromosomes in a single cell (1), exchange of plasmids or conjugative elements between different bacteria (2,3), movement of plasmids through multiple environments (4,5) and following microbes across continents (6,7). Each genome or metagenome sequence serves as a snapshot at a specific time and place. Comparing these snapshots allows us to identify closely related sequences across geographic and ecological boundaries, and track genetic information at different scales, from within a patient to across the globe.

Several mechanisms can lead to the repeated observation of a given microbial sequence. First, the observed strains may be very closely related, suggesting a recent physical exchange between the sampling sites, or both from a third source (8–10). Second, the sequence may reflect a conserved mobile genetic element (MGE), which is transferred between different microbial lineages independently of vertical inheritance (11). Either way, the timescale of the events may be inferred from the similarity between the two sequences. Just after such events the sequences should be highly similar, but over time they increasingly accumulate mutations such as point mutations, loss of segments, and insertion of new information via recombination (11,12). From the perspective of a given query, longer sequence matches reflect more recent exchanges. Thus, searches for the longest stretch of sequence identity in a database of target sequences can be used to identify the closest genomic link of the query.

Given the expanse and rates of exchange of DNA, especially in prokaryotes (13,14), there can be different matches across a single chromosome. To establish such links, chromosome painting has been used within cells and across species. Originally, chromosome painting was performed experimentally, by probing specific regions of the chromosome with fluorescently labeled DNA (15), and the resulting color patterns were used to compare chromosome structures. In case of genomic exchange or chromosomal rearrangements, the sequence segments can be traced back to their source based on their color. Experimental chromosomal painting has been applied to a variety of species such as plants (16,17) and fungi (18).

*In silico* chromosome painting similarly identifies genomic links between a query sequence and reference database, and has been used to study the population structure in, e.g. humans (19) and *Helicobacter pylori* (20). From a genomic forensics perspective, identifying the longest identical match of a given query segment would yield the closest genomic link, within the search frame of a given reference database. For example, in the case of a recent geographic migration or HGT, the best match might reflect the most likely origin or donor of a subsequence. Identical matches between two sequences are commonly defined as maximal exact matches (MEMs), which cannot be extended without introducing mismatches (21). Algorithms to identify MEMs can broadly be categorized into those using suffix arrays and their derivatives (21–24), and those that use hashing (25,26). Hashing is the most common solution to the MEM-finding problem used by tools such as E-MEM (25), bfMEM (26), and CopMEM2 (27). E-MEM utilizes hash tables to store k-mers from the reference genome, enabling rapid searches without the need for full-text indexing such as suffix arrays, which significantly reduces memory requirements and allows for efficient parallel processing. It can compute all MEMs of a minimum length of 100 between large genomes, such as human and mouse, in about 10 min using only 2 GB of RAM. In contrast, bfMEM employs a Bloom filter to manage k-mer data probabilistically, allowing for space-efficient storage and quick membership queries, although it may introduce false positives that require verification. In contrast to E-MEM, bfMEM only samples k-mers from the queries. CopMEM2 enhances the search for MEMs by utilizing a multithreaded approach and a specialized query data structure, achieving high performance on large datasets, such as human genomes, in a fraction of the time compared to its predecessors. These tools have shown excellent performance in resolving pairwise MEMs between a query and a large reference dataset. Still, pairwise comparisons of thousands of genomes remain computationally infeasible and result in large output files.

One data structure allowing efficient large-scale sequence comparison is the colored de Bruijn graph (cDBG). To construct a cDBG all the sequences are divided into k-mers that are used to build a de Bruijn graph (DBG), where edges represent the overlaps between adjacent k-mers in the sequences. Unlike DBGs, cDBGs also encode in what input sequence each k-mer, or node, was present, often referred to as the ‘color’ of the node. The reasonable k-mer size greatly depends on the question but generally ranges from 18 to 31 nucleotides (28). Each sequence used to build the cDBG traverses a specific path of nodes that overlaps with other paths in regions of sequence identity. To compress the cDBG, non-branching paths are merged into unitigs, resulting in a compacted cDBG (ccDBG) whose nodes are equal to or bigger than the original k-mer size. The compression feature of ccDBGs, combined with their ability to enable fast sequence comparisons, makes them suitable for tasks including sequence alignments (29), variant calling (30,31), homology detection (32), and genome-wide association studies (33). State-of-the-art ccDBG construction tools such as Cuttlefish and GGCAT (34), can process enormous amounts of sequences (35). Most graph builders produce output in Graphical Fragment Assembly format (GFA), which represents the graph and serves as a widely accepted input for tools utilizing ccDBGs. This promotes efficient data sharing by removing the need to build separate indexes for every tool, which is often the most time-consuming part (36).

Encoding a set of sequences into a ccDBG also implicitly resolves shared regions between the input sequences. For example, if two input sequences jointly traverse the same consecutive set of nodes, this indicates a shared identical substring or MEM. To identify MEMs for any given query, its path can be checked for intersections with paths of other sequences. This does require that the ccDBG is built from both the query and references simultaneously. In a large database, one query region could yield MEMs with thousands of different reference sequences, while we are often only interested in the longest match overall. Here we developed Graphite to find the longest MEM (LMEM) for each starting position in the query using a ccDBG. Resolving these LMEMs locally across a query provides unique insights into the closest genomic links that lie hidden in the wealth of matches. We demonstrate this by applying Graphite to a collection of *Campylobacter* genomes where we zoom in on two LMEMs that suggest an inter-species transfer of genetic material between *C. coli* and *C. jejuni* and between *C. coli* and the emerging pathogen *C. hyointestinalis* subsp. *Hyointestinalis* (37).

## Materials and methods

We describe the Graphite algorithm which finds the longest maximum exact matches (LMEMs) between one or multiple queries and a reference database. Graphite uses compacted colored de Bruijn graphs (ccDBGs) (38) and suffix arrays (SAs) (39) to discover LMEMs. These data structures are briefly described in the Supplementary Information.

### Defining the colored de Bruijn graph and LMEMs

Before we explain the algorithm, we first introduce nucleotide sequence data, the ccDBG and related terminology. Genomes are nucleotide sequences. Formally, these are represented as strings over the finite alphabet Σ={A,C,G,T}. Genomes can consist of one or more contiguous strings (contigs). Let $x$ be a string over $\Sigma$, that is $x \in {{\Sigma }^*}$, of length $| x |$ nucleotides, for which $x[ i \dots j]$ denotes a substring from positions $i$ to $j$, inclusive. We use a 1-based indexing with inclusive end points for ranges in line with the programming language Julia that was used to implement Graphite. $pre_l( x )$ and $suf_l(x)$ denote the prefix and suffix of $x$ with length $l$ such that $pre_l( x ) = x[1 \dots l]$ and $suf_l( x ) = x[| x | - l + 1 \dots | x | ]$. For any $x \in {{\Sigma }^*},$ its reverse complement $rcDNA\ ( x ) = \overline{x}$ is obtained by reversing $x$ and complementing each character based on nucleotide complementarity. The canonical form ${\boldsymbol{\hat{x}}}$ of a string is the lexicographically smallest of $x$ and $\overline{x}$, that is $\boldsymbol{\hat{x}} = \boldsymbol{\min} ( \boldsymbol{x}, \boldsymbol{\overline{x}} )$. Let $S = \{ {{{g}_1},\ {{g}_2},\ \ldots, \ {{g}_a}} \}$ be a collection of $a$ genomes where each genome ${{g}_i}$ can contain multiple contigs. Let $G\ ( {N,E} )$ be a colored de Bruijn (cDBG) graph of $S$ where $G$ is the graph, $N$ the set of nodes, and $E\ \subseteq \ N\ \times \ N$ the set of edges. The node set consists of all canonical substrings of length $k$ in $S$, usually called k-mers. Each node in $N$ is referenced by a unique identifier $n$ for which its canonical sequence can be obtained via $seq\ ( n ):\ {{\Sigma }^*}$. For any substring, $e$, of length $| e |\ = \ k\ + \ 1$ nucleotides there is an edge between the canonical k-mers that form the prefix and suffix of $e$. That is, the nodes $z$ and $o$, where $seq\ ( z ) = \widehat {{\boldsymbol{pr}}{{{\boldsymbol{e}}}_{\boldsymbol{k}}}{\boldsymbol{\ }}( {\boldsymbol{e}} )}{\boldsymbol{\ }}$ and $seq\ ( o ) = \widehat {{\boldsymbol{su}}{{{\boldsymbol{f}}}_{\boldsymbol{k}}}{\boldsymbol{\ }}( {\boldsymbol{e}} )}$, are connected by a directed edge $(z,o)$. Each contig, $c$, follows a walk $w$ over nodes and directed edges in $G$ that spell the contig string. We define a walk $w$ consisting of $| w |$ nodes as $w = [\ {{(n,d)}_1},\ ( {n,d{{)}_2},\ \ldots,\ ( {{{n}_{| w | - 1}},\ {{d}_{| w | - 1}}} ),\ ( {{{n}_{| w |}},\ {{d}_{| w |}}} )} ]$ where ${{n}_i}$ is the node identifier for position $i$ in the walk and ${{d}_i}\{ { + , - } \}$ defines the node sequence travel direction, where ‘$ +$’ indicates the canonical sequence of $n$, that is $seq\ ( n )$, and ‘$ -$’ the reverse complement of the canonical sequence of $n$, that is $\overline{\text{seq}(n)}$. For example, consider a graph with $k = 3$, and let $seq\ ( {{{n}_1}} )\ = \ ATG$ and $seq\ ({{n}_2})\ = \ CCA$. Then the walk $w = \{ {{{n}_1} + ,\ {{n}_2} - } \}$ would traverse $[ {ATG,\ \overline {CCA} ] = [ATG,\ TGG} ]$ spelling ATGG. We also define $rcWalk\ ( w )$ which reverses the order of the nodes and their directions. $rcWalk\ ( w ) = [\ {{(n, - d)}_{| w |}},\ {{(n, - d)}_{| w | - 1\ }},\ \ldots,\ {{(n, - d)}_2},\ {{(n, - d)}_1}$ where $ - d$ inverts the traversal direction. For example, if $w = [\ {{(n, + )}_1},\ ( {n, - {{)}_2}} ]$, then $rcWalk\ ( w )\ = [\ {{(n, - )}_2},\ ( {n, + {{)}_1}} ]$. We use $w[ {i \dots j} ]$ to indicate a sub-walk with $i$ and $j$ being the positions in $w$.

**Figure 1.**
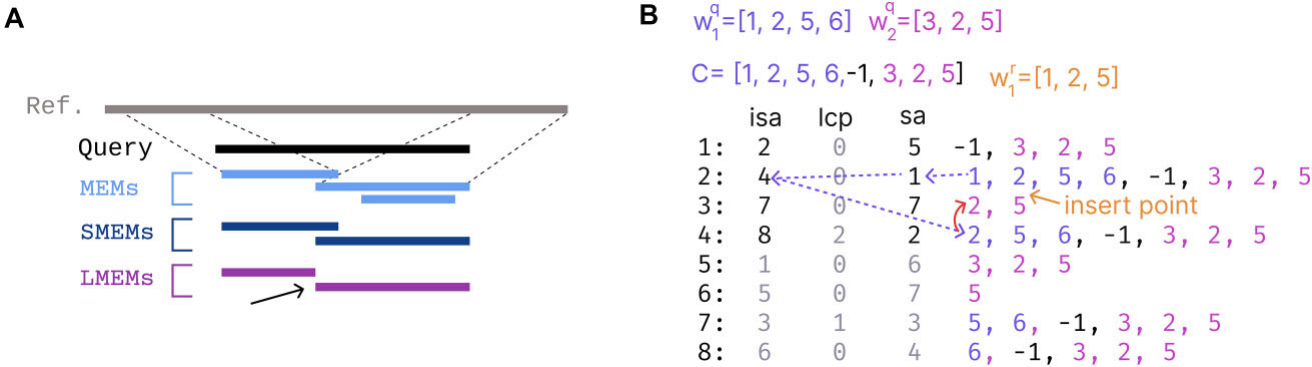
(**A**) LMEMs and suffix array queries. Formally, MEMs (Maximal Exact Matches) are matches between sequences that cannot be extended in either direction without encountering mismatches. SMEMs (Super Maximal Exact Matches) are a subset of MEMs that are not entirely contained within any other MEM. Similar to SMEMs, LMEMs (Longest Maximal Exact Matches) are not contained within any other MEM but additionally LMEMs do not overlap with other MEMs. When two SMEMs overlap, the overlapping region is assigned to the longer MEM, forming the LMEMs (black arrow). (**B**) This example demonstrates querying a reference walk, $w_1^r = [ {1,2,5} ]$ (orange) in a suffix array of two query walks, $w_1^q = [ {1,2,5,6} ]$ (purple) and $w_2^q = [ {3,2,5} ]$ (pink), where $C$ is the concatenation of both walks separated by a negative identifier (−1). The Longest Common Prefix array (LCP) stores the length of the shared prefix between two adjacent suffixes in the SA. The inverse SA (ISA) tell us, for each suffix in $C$, where it is located in the suffix array. For example, the first suffix (C, index = 1) is at index 2 in the suffix array according to the ISA, as $isa[ 1 ] = 2$. A binary search of the first suffix of $w_1^r$, that is [1,2,5] in $C$ locates a prefix match at index 2 with $w_1^q$, being [1,2,5] (insert point arrow). Matching the prefix [1,2,5] we know that all sub-suffixes in the prefix also exist as matches between C and $w_1^r$, that is [2,5] and [5] (not shown). Using a combination of the LCP and ISA we can directly identify other prefix matches between any query and suffixes of $w_1^r$. Following the ISA, according to ISA[SA[2]+1]=ISA[1 + 1]=ISA[2]=4, we first locate another prefix match [2,5] with $w_1^q$ at index 4. Using the LCP (red arrow) we also locate a match between $w_1^r$ and $w_2^q$. This is described in detail in the section ‘Reference Alignment’ and Algorithm 2.

To reduce data storage, Cuttlefish compresses the cDBG to a compacted cDBG (ccDBG) by collapsing nodes in non-branching walks into single nodes (35). Any ambiguity characters in the input sequences are not indexed, leading to potential breaks in the contigs. Consequently, the length of a canonical string for a $n\ \in \ N$ can now exceed $k$, especially if the sequences in the graph are highly similar. Before looking at LMEMs we first define when a query string $q\ \in \ S$ and a reference string $r\ \in \ S$, share a maximum exact match (MEM). Thus, $q$ and $r$ follow a walk through $G$, defined as ${{w}^q}$ and ${{w}^r}$, respectively. We can define a MEM, $m$, as a quadruple $m = ( {{{f}_q},\ {{t}_q},\ {{f}_r},\ {{t}_r}} )$ where $f$ and $t$ indicate the start and end positions in ${{w}^q}$ and ${{w}^r}$ such that ${{w}^q}[ {{{f}_q} \dots {{t}_q}} ] = {{w}^r}[ {{{f}_r} \dots {{t}_r}} ]$, ${{w}^q}[ {{{f}_q} - 1} ]\ \ne \ {{w}^r}[ {{{f}_r} - 1} ]$ and ${{w}^q}[ {{{t}_q} + 1} ]\ \ne \ {{w}^r}[ {{{t}_r} + 1} ],$ if defined, with $| m |$ being the length in nodes, such that $|m| = {{t}_q} - {{f}_q} + 1$ nodes. The length in nucleotides for a MEM is the sum of the node lengths excluding their overlap, defined as $nts:\ m\ \to \ \Sigma _{i = {{f}_q}}^{{{t}_q}}| {seq\ ( {n{{\ }_i}} )} |\ - \ ( {| m |\ - \ 1} )\ ( {k - 1} )$ where $| {seq\ ( {{{n}_i}} )} |$ is the length of the canonical sequence of ${{n}_i}$ in ${{w}_q}$ and ($| m |\ - \ 1)\ ( {k - 1} )$ is the number of nodes multiplied with the $k - 1$ to account for their overlap in the graph. MEMs describe a match between a single $q$ and a single $r$. In the case of LMEMs, we for each node in ${{w}_q}$ select the longest MEM, based on nucleotides, covering the node. Let $\mathcal{M}$ be the set of all $b$ MEMs between $q$ and all references, $\mathcal{M} = \{ {{{m}_1},\ {{m}_2},\ \ldots,\ {{m}_{| b | - 1}},\ {{m}_{| b |}}} \}$. All MEMs covering the ${{j}^{th}}$ node in ${{w}^q}$ are defined as ${{\mathcal{M}}_j} = \{ \mathcal{M}|{{f}_q}\ \le \ j\ \le \ {{t}_q}\}$. The LMEM for the ${{j}^{th}}$ node, ${{L}_j}$ is then ${{L}_j} = _{\ m \in {{M}_j}}^{arg\ max\ nts\ ( {{{m}_j}} )}$. There could be multiple, equally long MEMs in ${{L}_j}$. In this case we only retain any LMEM. The property of having only a single match per query region differentiates LMEMs from Super Maximal Exact Matches (SMEMs (40)), which serve as initial matches between queries and references to extend alignments from (Figure 1A).

**Table:** 

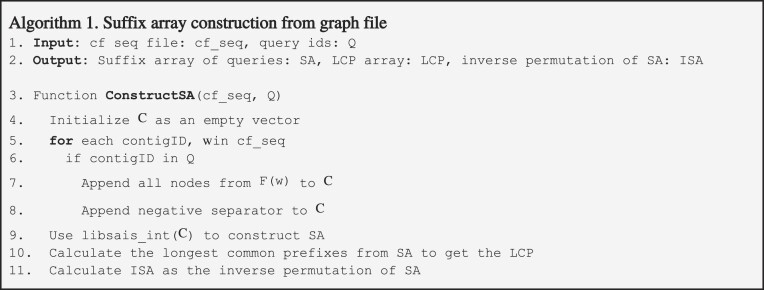

Given a node walk for the query, the LMEM finding problem is then to find all LMEMs with a set of references from the same graph. These steps are described in Algorithm 1.

### Algorithm overview

As input, Graphite takes a reduced GFA output produced by Cuttlefish 1 and a set $Q$ consisting of $| Q |$ query contig identifiers. Compared to Cuttlefish 2, the first version outputs the walks for each input sequence. The reduced GFA output consists of two files:

- The cf_seq file, which defines the contig walks;
- The cf_seg file, that contains node identifiers with corresponding canonical nucleotide sequences from which the previously introduced mapping $seq\ ( n ):\ {{\Sigma }^*}$ is derived.

Let the walks for the queries be denoted as ${{W}_Q} = [ {w_1^q,\ w_2^q,\ .,w_{| Q | - 1}^q,w_{| Q |}^q} ]$, where $w{{_i^q}^{}}$ is the walk for the ${{i}^{th}}$ contig in $Q$. A walk is defined as previously described, with canonical node sequences from the cf_seg file and their traversal direction from the cf_seq file. Taking the reduced GFA and Q as inputs, Graphite executes three phases: query SA construction, reference alignment and output generation.

### Query suffix array construction

Similar to bfMEM, we construct the SA from queries instead of references. For practical implementation, we constrain the node identifiers to 32-bit integers, using the least 30 significant bits to represent the node identifier. The 31st bit (second-highest) is reserved to indicate the travel direction and the role of the 32nd bit, or sign bit, is explained below. Node identifiers in the graph can fill all 32 bits, in the algorithm however $N\ = \ \{ n\ \in \ \mathbb{Z}\ |\ 0\ \le \ n\ \,<\, \ {{2}^{30}}\ - \ 1\}$ which is validated when reading the cf_seg file. To encode the traversal direction, if a node is traversed in its canonical form the second-highest bit of the node identifier is 0, otherwise 1. Let $F$ be the function that encodes the traversal directions for a walk $w$ defined as $\ F\ ( w ) = \ [{{v}_1},{{v}_2},\ \ldots,\ {{v}_{| w | - 1}},{{v}_{| w |}}]$ where $v_i = \{n_i \text{ if } d_i = + \text{ else } \text{flip}(n_i)\}$. Here, ${{n}_i}$ is the original node identifier, ${{v}_i}$ the node identifier with the second-highest bit set based on the traversal direction, and $flip$ a function that flips the second-highest bit. To construct the SA later, we apply $F$ to all query walks and concatenate them separated by negative identifiers. As every $v\ \in F( w )$ is unsigned (32nd bit is 0), negative separators do not affect the sorting of the suffixes later, and serve to separate different walks and identify the corresponding query contig identifier. We denote this concatenation vector as $C\ = [ {F\ ( {w_1^q} ) \odot ( { - 1} )\ \odot \ F\ ( {w_2^q} ) \odot \ ( { - 2} )\ \ldots\ \odot F\ ( {w_{| Q | - 1}^q} ) \odot}$ $ {( { - | Q | - 1} ) \odot F\ ( {w_{| Q |}^q} )\ \odot \ ( { - | Q |} )} ]$ with length $| C |$ (Figure 1B). Here, $ \odot$ represent the concatenation operation. The vector $C$ is used to construct the suffix array $sa$ which holds all the suffixes of $C$ in lexicographical order. Here the ${{i}^{th}}$ suffix is referred to as $C[i\dots]$. We also make use of the longest common prefix (LCP) array, referred to as $lcp$, and the inverse SA (ISA) referred to as $isa$. The LCP stores the length of the shared prefix between two adjacent suffixes in the $SA$. That is $lcp[ i ]$ is the shared prefix length between $sa[ i ]$ and $sa[ {i - 1} ]$. For $sa[ 1 ]$ there is no predecessor, hence $lcp[ 1 ]\ = \ 0$. The ISA maps each index $j$, ranging from 1 to $| C |$, in $C$ to the corresponding suffix index the suffix array. For example, $isa[ 1 ]$ gives the lexicographically ordered position of the suffix starting at position 1 (Figure 1B). The $\ C$, $sa$, $isa$, and $lcp$ arrays all have the same length. After constructing the SA, the LCP and ISA can be derived using standard algorithms (22). The steps to construct the data structures from a graph file are given in Algorithm 2.

**Table:** 

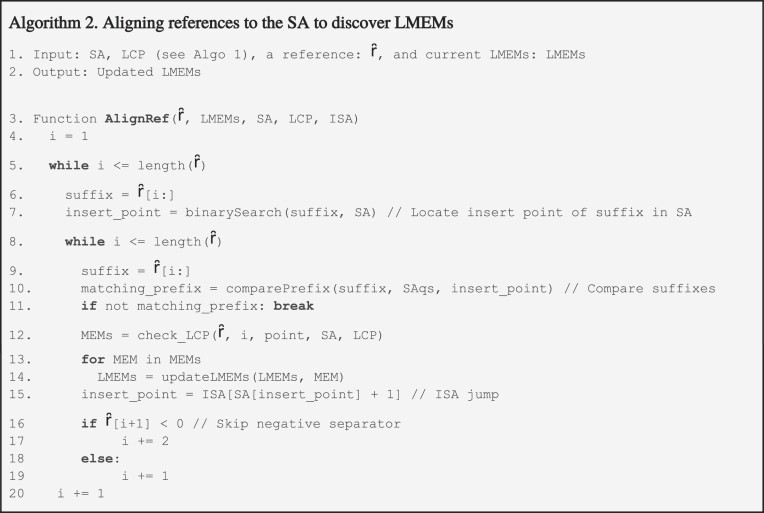

### Reference alignment

After constructing the SA of queries, the next step is to align the references to identify the LMEMs. The references are the walks for all contigs that are not in $Q$, formally $R = S\backslash Q$ with the total collection of reference walks being ${{W}^R} = \{ {w_1^r,\ w_2^r,\ \ldots,\ w_{| R | - 1}^r,\ w_{| R |}^r} \}$ where $| R |$ is the number of contig identifiers. Like for the queries, $F$ is applied to ${{W}^R}$, $F\ ( {{{W}^R}} )\ = [ {F\ ( {w_1^r} ),\ F\ ( {w_2^r} ),\ \ldots,F\ ( {w_{| R | - 1}^r} ),\ F\ (w_{| R |}^r} )]$. For simplicity we refer to any reference walk from $F\ ( {{{W}^R}} )$ as ${\mathrm{\overset{\scriptscriptstyle\frown}{r}}}$, and to its *j*th suffix as ${\mathrm{\overset{\scriptscriptstyle\frown}{r}}}[ {j \dots } ]$. We can discover a MEM, $m$, as shared prefix between a suffix from $C$, $C[ {i\dots} ]$, and suffix from ${\mathrm{\overset{\scriptscriptstyle\frown}{r}}}$, ${\mathrm{\overset{\scriptscriptstyle\frown}{r}}}[ {j \dots } ]$ as previously defined. Thus, MEM candidates can be identified by performing a binary search of every suffix in ${\mathrm{\overset{\scriptscriptstyle\frown}{r}}}$ in $C$. However, this is an redundant approach which potentially repeatedly recomputes prefix matches. We use several tricks to speed up the discovery of prefix matches, and thus potential MEMs, between references and $C$, specifically on two occasions. First, we use the LCP to quickly obtain MEMs that share the prefix, or sub-prefix with a previously located MEM (Figure 1B, red arrow). When a binary search finds a MEM, $m$, at location $t$ in the SA we can directly scan up ($t - 1)$ and down ($t\ + \ 1$) in the SA for other suffixes of $C$ also matching ${\mathrm{\overset{\scriptscriptstyle\frown}{r}}}[ {j \dots } ]$. Namely, if $lcp[ t ]\ >\ 0$, the suffix at $SA[ {t - 1} ]$ also shares a prefix with ${\mathrm{\overset{\scriptscriptstyle\frown}{r}}}[ {j \dots } ]$ of at least length $min\ (| m |, lcp[t])$ nodes. Similarly, if $lcp[ {t + 1} ]\ >0$, $SA[ {t + 1} ]$ shares a prefix of at least $min\ (| m |, lcp[t])$. Either side can be investigated until $lcp[ t ]\ = 0$, $i = 1$, $j = 1$, $i\ = | C |$, or $j = | C |$ (‘check_lcp’ in Algorithm 2). This eliminates a lot of costly node comparisons as the shared prefixes do not have to be repeatedly computed. The second speed up comes from utilizing the ISA (Figure 1b, purple arrows). When discovering a MEM of $| m |\ >\ 1$, we know that the second suffix in ${\mathrm{\overset{\scriptscriptstyle\frown}{r}}}$, ${\mathrm{\overset{\scriptscriptstyle\frown}{r}}}[ {j + 1 \dots } ]$ and the second suffix in $C$, $C[ {i + 1 \dots} ],$ also share a prefix of length $| m | - 1$ nodes. Combining this with the LCP scan above, if we could locate $C[ {i + 1 \dots} ]$ in the SA we can directly jump to the SA location where potentially multiple shared prefixes with ${\mathrm{\overset{\scriptscriptstyle\frown}{r}}}[ {j + 1\dots} ]$ exist without performing a binary search. Locating $C[i + 1\dots]$ in the SA is facilitated by the ISA as $C[ {i + 1 \dots} ]$ is at position $ISA[ {SA[ k ] + 1} ]$ in the SA, such that $C{{[ {i + 1\dots} ]}_{}} = \ C[ {ISA} [SA[ k ] + 1] \dots ]$ (Figure 1b, Supplementary Figure S1). Thus, combining the LCP and ISA can prevent costly binary searches and node comparisons. To find reverse complement matches we only have to reverse the order of the nodes in each walk and invert their traversal directions. Formally, for each ${\mathrm{\overset{\scriptscriptstyle\frown}{r}}} = [ {{{v}_1},\ {{v}_2},\ \ldots ,\ {{v}_{| {{\mathrm{\overset{\scriptscriptstyle\frown}{r}}}} | - 1}},\ {{v}_{| {{\mathrm{\overset{\scriptscriptstyle\frown}{r}}}} |}}} ]$ in $F\ ( {{{W}^R}} )$ we create its reverse complement as follows: $flip\ ( {{\mathrm{\overset{\scriptscriptstyle\frown}{r}}}} ) = [ {flip\ ( {{{v}_{{\mathrm{\overset{\scriptscriptstyle\frown}{r}}}}}} ),\ flip\ ( {{{v}_{| {{\mathrm{\overset{\scriptscriptstyle\frown}{r}}}} | - 1}}} ),\ \ldots ,\ flip\ ( {{{v}_2}} ),\ flip\ ( {{{v}_1}} )} ]$. Referring to the reverse complement of all reference walks $flip\ {{(F\ ({{W}^R}))}_{}}$we apply the above described steps to both $F\ ( {{{W}^R}} )$ and $flip\ {{(F\ ({{W}^R}))}_{}}$ to discover all MEMs between the contigs from $Q$ and $R$. Given multiple MEMs we can then select LMEMs as defined previously. The steps are summarized in Algorithm 2.

### Updating LMEMs

In our Algorithm 2 description, we omitted details on optimizing LMEM updates (Algorithm 2: line 14). We have two vectors of length $| C |$, $LME{{M}_{size}}$ and $LME{{M}_{origin}}$. The former stores the LMEM size (in nucleotides) of each node in the MEM, and the latter the reference identifier and MEM start position in $C$. Initially both hold zeros, $LME{{M}_{size}} = [ {{{0}_1},{{0}_2}{{{, \dots ,0}}_{| C | - 1}},{{0}_{| C |}}} ]$ and $LME{{M}_{origin}} = [\ {{(0,\ 0)}_1},\ {{(0,\ 0)}_2},\ \ldots,\ {{(0,0)}_{| C | - 1}},\ ( {0,0{{)}_{| C |}}} ]$. The start position is required to distinguish different LMEMs from the same reference. We will use an example to clarify. Let ${{m}_1}$ be a MEM of length $nts\ ( {{{m}_1}} )\ = \ 2$, originating from the prefix $C[ {1.2} ]$ with the reference contig identifier ‘$1$’. The updated arrays would be $LME{{M}_{size}} = [ {{{2}_1},{{2}_2},{{0}_3},\ \ldots,\ {{0}_{| C | - 1}},\ {{0}_{| C |}}} ]$ and $LME{{M}_{origin}} = [\ {{(1,1)}_1},\ {{(1,\ 1)}_2},\ {{(0,0)}_{3\ }},\ \ldots,\ {{(0,0)}_{| C | - 1}},\ ( {0,0{{)}_{| C |}}} ]$. Then a second MEM, ${{m}_2}$, is discovered with $nts\ ( {{{m}_2}} ) = 3$ covering $C[ {2.4} ]$ with reference ‘$7$’. As ${{m}_2}$ with length 3 is longer than ${{m}_1}$ we overwrite any overlaps with ${{m}_1}$. That is, $LME{{M}_{size}} = [ {{{2}_1},{{3}_2},\ {{3}_3},\ {{3}_4},{{0}_5},\ \ldots,\ {{0}_{| C | - 1}},\ {{0}_{| C |}}} ]$ and $LME{{M}_{origin}} = [ {{(1,1)}_1}, {{(7, 2)}_2}, {{(7,2)}_{3 }}, {{(7,2)}_{4 }}, {{(0,0)}_5}, \ldots, {{(0,0)}_{| C | - 1}}, ( {0,0{{)}_{| C |}}} ]$. This allows us to quickly check whether we previously discovered a longer MEM. Given a MEM $m$ for the prefix $C[ {i \dots | m |} ]$, if $ME{{M}_{size}}[ i ]\ >nts\ ( m )\ \& \ ME{{M}_{size}}[ {| m |} ]\ > \ nts\ ( m )$ we have an existing longer MEM so we do not have to check every position from $i\ .| m |$ in $ME{{M}_{size}}$.

### Parallelization of reference alignment

All suffixes of ${\mathrm{\hat{r}}}$ can be compared to the SA using multiple threads, parallelizing Algorithm 2 line 5. For this we equally split ${\mathrm{\hat{r}}}$ based on the number of provided threads. As suffixes in different threads can yield a MEM covering the same position $i$ in $C$, and thereby target to simultaneously modify $Origi{{n}_{LMEM}}[ i ]$ and $Siz{{e}_{LMEM}}[ i ]$, we use atomic operations and spinlocks to prevent multiple threads updating the same LMEM.

### Graphite output generation

**Figure 2.**
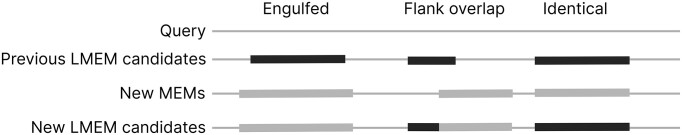
LMEM replacement criteria. Upon discovering a new MEM, we compare its length to the previous LMEM candidates covering the same sites. Three columns indicate different possibilities. If the new MEM is longer or if there is no previous LMEM at those sites, it replaces the current LMEM (engulfed). When the MEM and LMEM have overlapping regions, the overlapping region gets assigned to the longest of the two (flank overlap). If a MEM matches the LMEM’s length precisely, we retain the initially found LMEM (identical).

We generate a tab-separated file containing the query identifier, the reference identifier, the LMEM start position, the LMEM end position, and the original MEM size. As the original MEM is an SMEM that might be trimmed (Figure 2), its size compared to the LMEM size provides insight into the extent of trimming.

### Graphite validation and application to existing datasets

We collected all complete *Campylobacter* genomes, excluding those shorter than 1.5 MB, from BV-BRC (41) and built a MashTree (42) that was rooted on the ancestor of *C. coli* and *C. jejuni* and visualized with iTOL (43). We re-annotated species when their BV-BRC annotation deviated from that of the majority in the monophyletic clade (see Supplementary Table S1). To use Graphite, we built a ccDBG of all *Campylobacter* genomes with Cuttlefish (35) (k-mer size: 31) and saved storage space using reduced GFA (-f 3) output format.

LMEM selection by Graphite was validated on three *C. jejuni* genomes (CP071576.1, CP071584.1, CP071578.1) and compared to E-MEM results with match size 31 (‘-l’) while allowing matches on both strands (‘-b’). Correct LMEMs were determined by their maximum length among all MEMs covering a position as defined previously under ‘*Defining the colored de Bruijn graph and LMEMs’*.

To evaluate Graphite's performance, we compared it to E-MEM (25), bfMEM (26) and CopMEM2 (27). We queried *C. coli* genomes against all other *Campylobacter* genomes using all three tools. E-MEM used the same settings as earlier. For bfMEM we used a minimum match size of 33 (-l) and a k-mer size of 32 (-k) allowing matches on both strands (-s b). This choice was due to restrictions in bfMEM, requiring k%4 == 0 and l > k. CopMEM2 was restricted to MEMs of at least 50 nucleotides (-l 50) and MEMs on both strands were allowed (-b). The computer setup was consistent: Intel (R) Xeon (R) Platinum 8280L CPU @ 2.70 GHz CPU, 3TB RAM and an HDD storage drive.

To demonstrate Graphite's ability to efficiently detect DNA exchange, we applied it to all *Campylobacter* genomes, comparing each genome to the others, and highlighting striking examples of HGT. Directly visualizing interesting LMEM regions and their connections in the context of the full ccDBG could result in an explosion of paths and nodes, obfuscating the actual shared region and context. To simplify the visualization, we built a tool,subgraphs, to show a limited representation of the visualized LMEMs in the graph context. In Figure 6, for example, we visualized LMEMs using parameters *n* = 0.9 and *l* = 1000, where n determines the minimum fraction of nodes overlapping the LMEM for inclusion, and l specifies the number of extending nodes on each side. See https://github.com/mgxlab/subgraphs for the implementation. The simplified subgraph was then visualized using Bandage (44) and gene plots were created using DNA Features Viewer (45).

## Results

We developed Graphite, available at https://github.com/MGXlab/Graphite, to identify the longest maximum exact matches (LMEMs) between one or more queries and multiple reference sequences. Below, we first validate LMEM identification by comparing our results to MEMs identified by E-MEM. Then we use an example dataset composed of 576 *Campylobacter* genomes to show how LMEMs can drastically reduce the number of matches and thus the output file size, and demonstrate how they shed light on relevant evolutionary events.

### Validation of LMEM identification on three bacterial genomes

**Figure 3.**
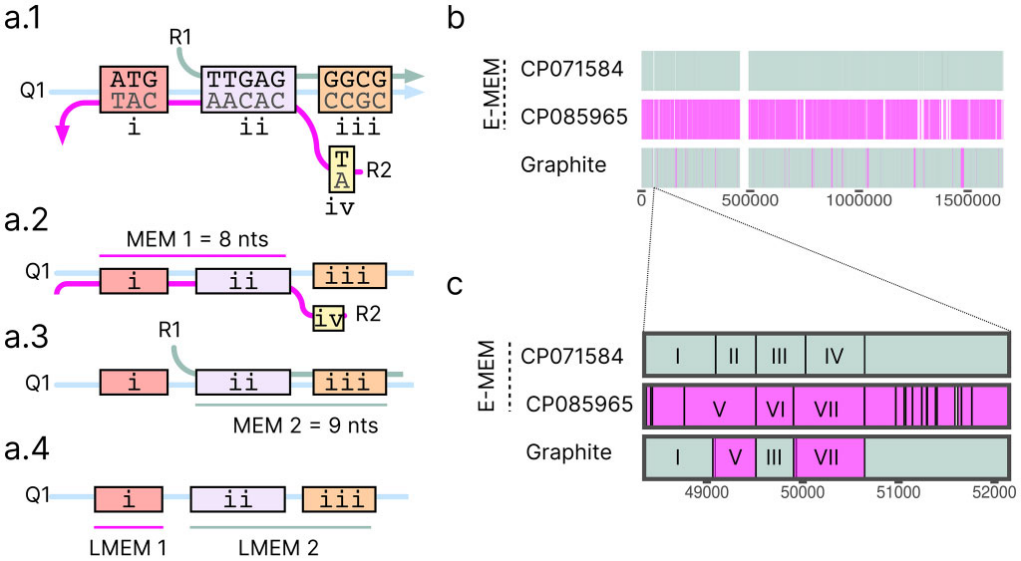
Longest maximum exact match (LMEM) selection. (**a**) In a compacted graph, the variation in the overall sequence set determines the sequence fragmentation and node size. **a.1** shows a toy graph of three sequences Q1, R1, and R2 that are composed of four nodes i–iv (see Supplementary Figure S2 for further details). The k + 1 overlap between nodes as seen in ccDBGs is not shown for simplicity. The sequence strands are indicated with arrows, the forward and reverse sequences are shown in the nodes. While Q1 and R2 share a MEM of 8nt (**a.2**) consisting of nodes i-ii, it does not show up as a single node since another sequence, R1 contains a subsequence of this MEM (node ii, **a.1**). Similarly, Q1 and R1 share a MEM of 9nt (**a.3**) consisting of nodes ii–iii. **a.4** shows how two LMEMs are resolved for this example. Each node is assigned to the longest MEM it is part of. Node ii is part of two MEMs and gets merged with node iii into LMEM 2 (9nt) which is 1 nucleotide longer than MEM 1 (8nt). As the remaining node i is only covered by MEM 1, it is assigned to LMEM 1, and the query sequence is painted accordingly (**a.4**). (**b**) To validate Graphite on real data we aligned *C. jejuni* CP071576 against CP071584 and CP085965 using E-MEM and Graphite. The majority of Graphite LMEMs originated from CP071584 (gray). (**c**) Close-up example of LMEM selection. First, MEM I is selected from CP071584 as it is longer than the multiple overlapping CP085965 MEMs. A part of MEM V is selected as the next LMEM, as V is longer than II. Likewise, a part of MEM III is selected over MEM VI before Graphite's LMEMs continue into VII.

Graphite uses a ccDBG as input, in this case, we built them using Cuttlefish with a k-mer size of 31 nucleotides (35). Node sizes in the ccDBG depend on the sequence variation in the input sequences. Mutations such as SNPs introduce bubbles in the cDBG and prevent merging adjacent k-mers into longer sequences during graph compaction. As nodes may be shared by all sequences that overlap by at least 31 nucleotides, a shared node rarely forms the entire MEM, as variations often occur in sequences other than those being compared (Figure 3a). Graphite locates the shared nodes between a query sequence and each reference to bidirectionally extend these resolving a MEM. As all references in the database are compared, a given query region will match multiple MEMs, of which Graphite only retains the longest. To validate LMEM selection by Graphite we used both E-MEM and Graphite to query *C. jejuni* CP071576 against the CP071584 and CP085965 genomes. E-MEM identified 1586 MEMs with CP071584, and 10 990 MEMs with CP085965. Graphite resolved 1564 LMEMs, most selected from CP071584 with some exceptions (Figure 3b). Figure 3c shows how these exceptions support Graphite's LMEM selection algorithm.

### Graphite efficiently identifies LMEMs in hundreds of genomes

To test Graphite on a medium-sized real world dataset, we downloaded all 579 complete *Campylobacter* genomes from BV-BRC (41). After curation 576 remained (see Online Methods, Supplementary Figure S3, Supplementary Table S1) which were used to construct a ccDBG using Cuttlefish (35) with 3 496 408 nodes and 115 691 195 edges. The genomes spanned 33 different species (excluding *Campylobacter* sp.) with the majority of genomes being derived from *C. jejuni* (*n* = 363, 63%) and *C. coli* (*n* = 86, 15%), while 29 species were represented by <10 genomes. Next, we compared the performance of different tools for finding MEMs including E-MEM (25), bfMEM (26), CopMEM2 (27) and LMEMs by Graphite, querying all 86 *C. coli* genomes (147 MB) against the remaining 490 *Campylobacter* genomes with a total size of 840MB (Table 1). In addition, we repeated the benchmark from the E-MEM paper (25) querying the whole human genome (3.2GB) versus the mouse genome (2.6GB) for Graphite and CopMEM2. CopMEM2, implemented in C++, was consistently the fastest tool among all. Graphite was 2–3x slower than CopMEM2 on smaller datasets but its output is only in the megabyte range compared to gigabytes for CopMEM2 and the other tools. For example E-MEM which we could run using the same k-mer size as Graphite produced an output file of 9.2GB compared to just 40 MB for Graphite. We note that the ccDBG only has to be constructed once and all sequences within the graph can then be used as a query. Moreover, the graph can serve as input for other analyses such as variant calling and visualization (30–32). By focusing on finding LMEMs we can significantly save storage space, especially when querying big reference datasets. As we are often only interested in the longest matches, for example for *in silico* chromosomal painting or HGT analysis, Graphite obsoletes post-processing of vast numbers of MEM results by already selecting the longest MEM during alignment.

**Table 1.** Performance comparison of Graphite and two MEM finding algorithms when querying 86 *C. coli* versus 490 other *Campylobacter* genomes using Graphite and two MEM finding algorithms

Method	Dataset	Result	Output size (GB)	Runtime (h:m:s)	Memory (GB)	Threads
Graphite	*Campylobacter*	566 097 LMEMs≥ 31nt	**0.04**	00:08:57^a^	**3.38**	1
E-MEM	*Campylobacter*	126 946 345 MEMs ≥ 31nt	9.20	00:10:16	9.63	1
bfMEM	*Campylobacter*	108 719 433 MEMs ≥ 33nt^b^	4.40	00:34:22	15.40	1
CopMEM2	*Campylobacter*	55 016 810^c^≥ 50nt	2.20	**00:03:18**	4.29	1
Graphite	*Campylobacter*	566 097 LMEMs≥ 31nt	**0.04**	00:03:27^a^	**4.86**	20
E-MEM	*Campylobacter*	126 946 345 MEMs ≥ 31nt	9.20	00:06:09	9.63	20
bfMEM	*Campylobacter*	108 719 433 MEMs ≥ 33nt^b^	4.40	00:05:54	15.33	20
CopMEM2	*Campylobacter*	55 016 810^c^≥ 50nt	2.20	**00:00:54**	5.39	20
Graphite	Human vs. mouse	4 169 580 LMEMs ≥ 31nt	**0.20**	02:44:57^a^	35.00	70
CopMEM2	Human vs. mouse	1 849 841 302 ≥ 50nt	49.00	**00:04:52**	11.00	64^d^

^a^Includes ccDBG construction of 1 min and 27 s on 20 threads for Campylobacter, and 2 min and 29 s for human versus mouse

^b^bfMEM only supports k-mers of k%4==0 and the minimum match length should be greater than k. The number closest to 31 divisible by 4 is 32, so the minimum MEM length is 33.

^c^Encountered the message ‘block sort failed, falling back to full sort’, and the minimum length of MEMs was 50.

^d^Threads for CopMEM2 are limited to the number of input sequences.

Runtimes were rounded to minutes.

### Discovery of unique nodes in the Graphite ccDBG reveals unexpected genomic entities

**Figure 4.**
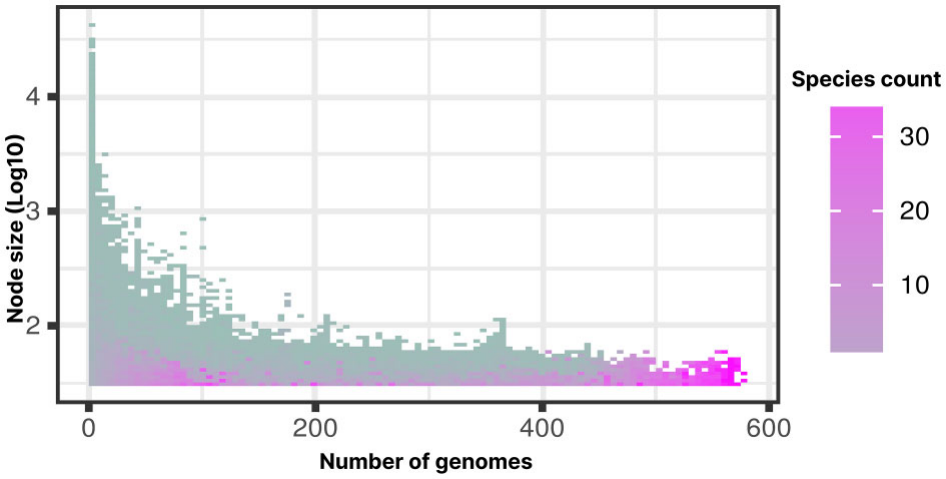
Relation between the size of nodes and the number of genomes and species they occur in. Short nodes may occur in many genomes and species, while long nodes over 120nt rarely occur in more than 100 genomes.

Besides that the ccDBG can be used to efficiently find MEMs, incorporation of the data into a graph structure also provides the opportunity to reveal unique genomic features. Generally, short nodes in the ccDBG were shared by more genomes than long ones (Figure 4). For example, nodes present in at least half of the genomes (≥288) were on average 64 nucleotides (nt) long with the longest being only 112nt. Of all 3.5 million nodes, 24.8%, 9.6% and 0.07% were present in at least 10, 100 and 500 genomes, respectively. The abundant sharing of the nodes between different genomes illustrates how nodes can function as anchor points for MEM and LMEM detection. The top five longest nodes in the graph exceeded 25 000nt and were all present in a single species. These large nodes represent non-branching paths in the graph, i.e. consisting of unique 31-mers that are never found in another genome in the dataset. They originated from *C. jejuni* (42 403nt), *Campylobacter* sp. (31 087nt) as well as under-represented species *C. curvus* (29 437nt, four genomes), and *C. concisus* (28 388nt and 27 886, eight genomes). The unique *C. jejuni* node corresponded to a sequence annotated as plasmid pFORC_083_2, found in *C. jejuni* strain FORC_083 that was isolated from chicken in South Korea. It is interesting to note that this plasmid probably represents a bacteriophage, as it encodes typical bacteriophage genes including structural and replication clusters, integrase, and a terminase large subunit that is related to sequences found in various *Firmicutes* (Supplementary Figure S4), while *Campylobacter* belongs to the *Epsilonproteobacteria* class. It is also predicted as a member of the *Caudoviricetes* class by IMG/VR (46) and was found by CRISPRimmunity (47) to match a 30nt CRISRPR spacer in the firmicute *Lactobacillus delbrueckii* subsp. *delbrueckii* strain KCTC 13731 with three mismatches (90% identity). Whether this is a case of contamination in this complete genome sequence or a bacteriophage with an unusually wide host range remains to be seen (48), but the example illustrates that the detection of long, unique nodes in a pan-genome graph may quickly reveal unexpected or foreign sequences for follow-up.

### LMEMs quantify interspecies genetic transfer within the *Campylobacter* genus

**Figure 5.**
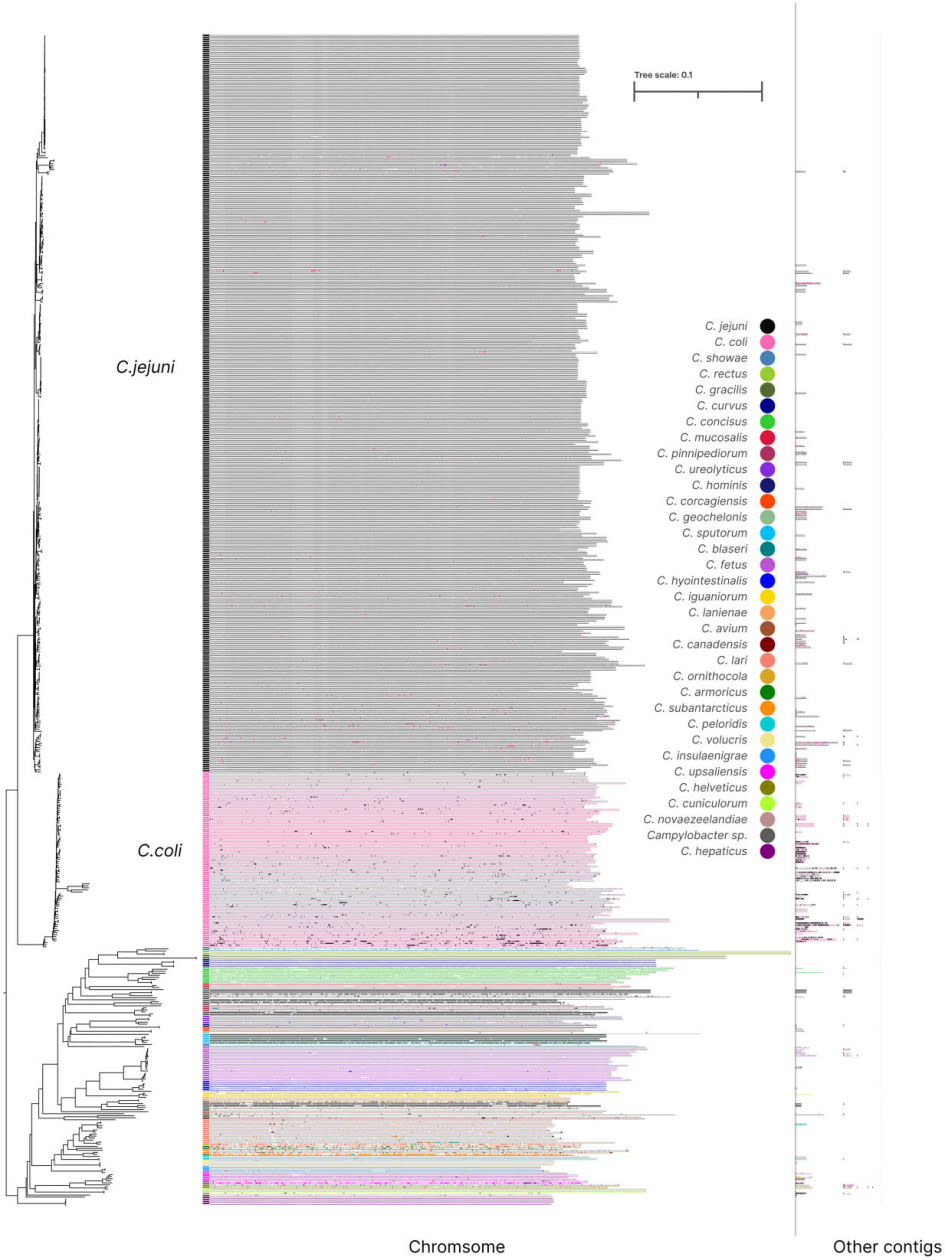
LMEM painting of 576 *Campylobacter* genomes. On the left is the Masthree clustering (Supplementary Figure S3). To the right, each row represents a query genome along a colored line representing the species it belongs to. Based on the LMEMs, the genomes are painted above the line, colored according to the matched species, or gray when the LMEM was found in the same species. For example, *C. coli* LMEMs matching *C. jejuni* are black in the *C. coli* clade, while *C. jejuni* LMEMs matching *C. coli* are pink in the *C. jejuni* clade.

*Campylobacter* is known to actively engage in horizontal gene transfer (HGT) via various mechanisms, such as the uptake of environmental DNA and the exchange of plasmids (49). To demonstrate how LMEMs can contribute to the detection of such genomic links and zoom in on long regions that are likely recently acquired, we queried each individual genome against all others using Graphite, which took 5 h and resulted in 2 109 030 LMEMs. One advantage of LMEMs is that they form a one-dimensional output containing, for each query region, the longest match in the database. This can be plotted to obtain a genome painting visualization for every genome in the *Campylobacter* genus, which reveals substantial HGT (Figure 5). When considering all LMEMs, 5.29% of the *C. coli* sequence had the closest match in *C. jejuni*, and 2.64% of the *C. jejuni* DNA had the closest match in *C. coli*. The frequencies of shared DNA between these two species varied greatly depending on the contig, ranging from 0 to 99.93% for *C. jejuni* and from 0.000112% to 99.81% for *C. coli*. While high frequencies were mainly observed for shorter contigs (Figure 5, Supplementary Figure S5), the *C. coli* chromosome CP092025 matched 19% of its DNA with *C. jejuni* and the *C. jejuni* chromosome CP059964 matched 10% of its DNA with *C. coli*. These results confirm the high transfer frequency of genetic material between *C. coli* and *C. jejuni* (50).

### LMEMs highlight biologically relevant genomic transfer events

**Figure 6.**
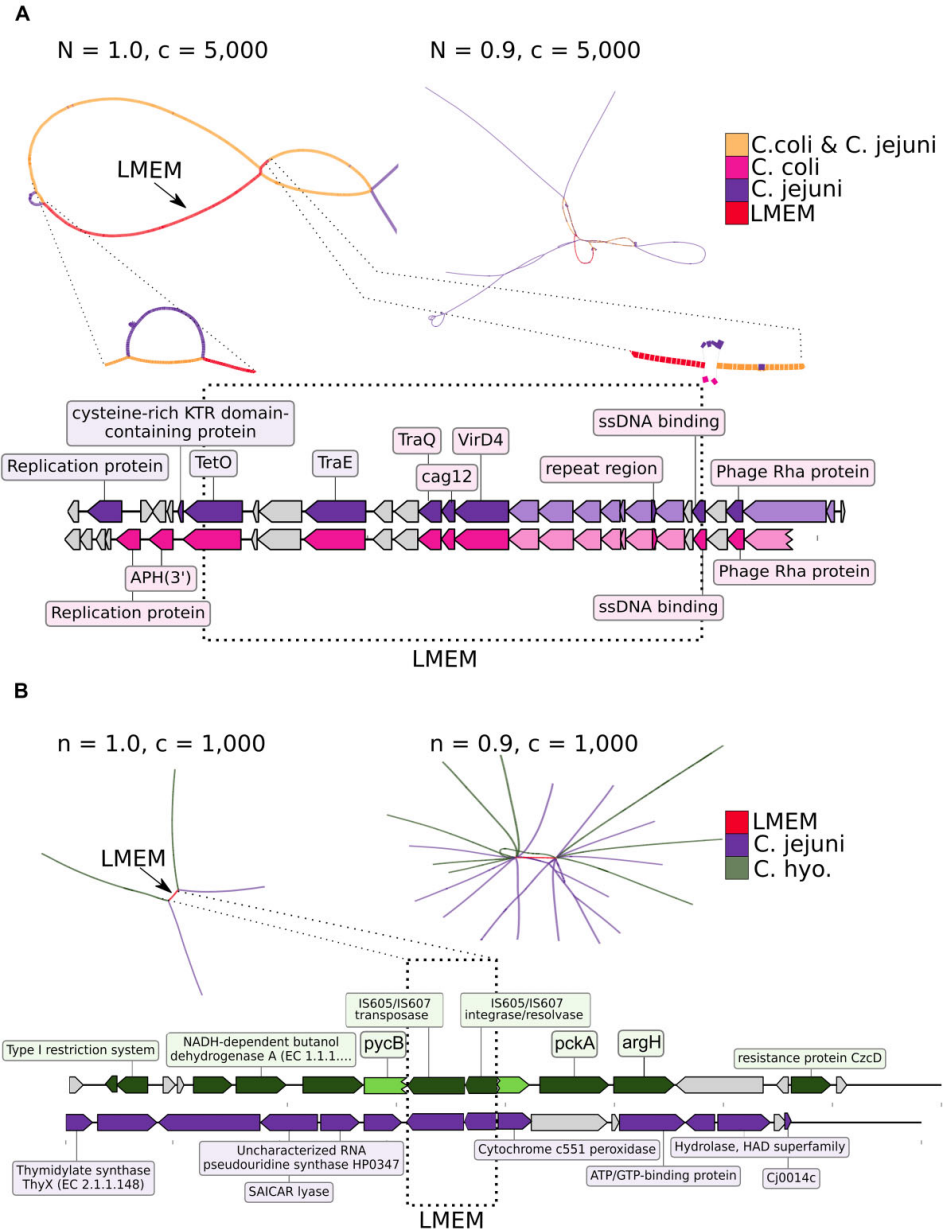
Example subgraphs of two LMEMs. (**A**) A 16 417nt LMEM between a *C. coli* plasmid (CP013035, positions 23 541–39 957) and *C. jejuni* chromosome (CP107256: 1 208 016–1 224 432). The parameter *c* specifies the fraction of LMEM nodes that a path has to traverse in order to be included in the subgraph visualization and n reflects the number of flanking nodes to display (see online methods). The left panel in A shows the subgraph of the LMEM (*n* = 1.0) with two genomes, 11 045 nodes and 11 057 edges. The right panel shows a relaxed subgraph of the LMEM with paths sharing ≥90% (*n* = 0.9) of the LMEM nodes. This relaxed subgraph contained 23 051 nodes and 23 665 edges, with paths from 18 genomes. In the left panel we zoom in on the bubbles that prevent further LMEM extension and for example, see that *C. coli* had aminoglycoside resistance gene (APH(3′)) flanking the LMEM which was absent in *C. jejuni* at this position. The gray-colored genes were annotated as hypothetical by BV-BRC and the light-colored genes encoded VirB proteins but their annotations were omitted for clearer visualization. (**B**) A subgraph with 4103 nodes and 4102 edges for a 2068nt LMEM encoding a transposon was found in two genomes from *C. jejuni* (CP047082: 58 822–60 889) and *C. hyointestinalis subsp. hyointestinalis* (CP015575: 1 258 714–1 260 781) abbreviated as *C. hyo*. For the latter species, the transposon disrupted the *pycB* gene encoding a pyruvate carboxylase according to the NCBI annotation (light green). When we slightly relaxed the subgraph threshold to *n* = 0.9 we obtained a subgraph with 20 255 nodes and 20 270 edges, with paths from 20 genomes. The extensive branching of paths outside the LMEM indicates the diverse genomic context of this transposon in different *C. jejuni* and *C. hyointestinalis subsp. hyointestinalis* genomes.

To further explore our results, we investigated long LMEMs that were shared by genomes from different species in search of striking cases of HGT. One LMEM of 16 417nt was shared between a *C. jejuni* chromosome (CP107256) and a *C. coli* plasmid (CP013035, Figure 6A). This LMEM encoded several proteins including the *tetO* gene conferring resistance against tetracycline antibiotics. Inter-species LMEMs were also observed among other species (Figure 5), for example, we detected a 2068nt LMEM between *C. jejuni* (CP047082) and *C. hyointestinalis* subsp. *hyointestinalis* (CP015575), which encodes an ISChh1 transposon according to ISfinder (51) that disrupted the *pycB* gene in *C. hyointestinalis* subsp. *hyointestinalis (*Figure 6B). We also explored other paths in the cDBG that traversed the LMEM nodes, showing that the transposon was present in twenty genomes in a variety of different contexts (Figure 6B). These plots can directly be generated from the Graphite output using our *subgraphs* tool available at https://github.com/mgxlab/subgraphs.

## Discussion

*In silico* chromosome painting allows source tracking of individual query sequences in high throughput. Here we present Graphite, an algorithm that efficiently identifies LMEMs between query and reference genome sequences by exploiting the ccDBG data format. Graphite addresses the challenge posed by traditional MEM finders, which often generate an overwhelming number of MEMs (25), complicating downstream analysis. For example, while filtering MEMs based on a minimum length can effectively reduce the number of matches, it could also exclude MEMs in regions that are inherently more variable. Graphite overcomes this by locally selecting a single LMEM, reducing the number of MEMs by over 30× compared to three established MEM finders (Table 1) while also retaining optimal query coverage (Figure 3). Moreover, on smaller datasets, Graphite is faster than bfMEM and E-MEM but slower than CopMEM2. Although CopMEM2 was the fastest across all datasets, it generally consumed more RAM than the other tools. It should be noted that we only tested this on a small subset of genomes, and performance might vary depending on factors such as the sequence similarity between the query and reference, and the size of the query and reference sequences in the graph. We anticipate that Graphite will excel when many similar queries are included in the graph, as the properties of the suffix array enable the rapid discovery of suffixes and sub-suffixes of nearly identical sequences. While suffix arrays were replaced by hashing for the MEM-finding problem, we believe that with advancements in graph compression, such data structures might become usable again without exhausting RAM, as node identifiers can be used instead of full sequences as done in Graphite. While Graphite used less RAM on smaller datasets, the hashing algorithms used less RAM on the larger human versus mouse comparison. With more optimizations in Graphite, such as the usage of sparse suffix arrays (21), we expect further improvements in both speed and memory efficiency. Moreover, the rapid advancements in graph builders, their compression, and query capabilities suggest that tools like Graphite, built on graph structures, offer a strong foundation for future discovery.

We showed that LMEMs not only serve as an output filtering approach but also provide new insights to answer biological questions. For example, by using Graphite to identify LMEMs in an all-versus-all comparison involving 576 *Campylobacter* genomes, we quantified overall inter-species genomic transfer rates and highlighted interesting cases. Notably, inter-species LMEMs were particularly prevalent among shorter contigs (on the right in Figure 5), which likely represent plasmids or other mobile genetic elements (MGEs) that are extensively shared within the *Campylobacter* genus (49). We also found that inter-species LMEMs between *C. coli* and *C. jejuni* were common for chromosomal contigs sometimes covering up to 19% of the sequence. Interestingly, our LMEM analysis also revealed that *C. coli* shared a greater portion of its DNA with *C. jejuni* than vice versa, which is in line with previous observations that suggest a directionality in the transfer between these species (52). *C. jejuni* was overrepresented in our dataset which might have affected this observation. The abundant exchange of genetic material between *C. coli* and *C. jejuni* has been proposed to result in a process of introgression or despeciation (52).

Our results support the notion that LMEMs can effectively identify inter-species transfers. For example, we identified a likely bacteriophage sequence in *C. jejuni* that was previously associated to *Firmicutes*. Zooming in on *Campylobacter*, we found a 16,417nt tetracycline resistance encoding LMEM between a 44 064nt *C. coli* plasmid and *C. jejuni* chromosome (Figure 6A). In addition, our subgraph visualization of the LMEM region showed that almost all nodes of the *C. coli* plasmid were present in the *C. jejuni* chromosome. Previously, this particular *C. jejuni* chromosomal segment had been theorized to arise from a conjugation event, wherein a *tetO* gene and a prophage were introduced into a plasmid, possibly from a common ancestor (53). The fact that Graphite linked this *C. jejuni* region as an LMEM to a *C. coli* plasmid suggests that *C. coli* played a role in this event as a recent donor or recipient of the sequence from *C. jejuni* (Figure 6B). Another example is the ISChh1 transposon-encoding LMEM between *C. jejuni* and *C. hyointestinalis subsp. hyointestinalis* (Figure 6B). While horizontal gene transfer between these two species is not extensively researched, observations suggest that they engage in recombination when cohabiting in the same environment (54). Although we did not observe it in this instance, transposons, including ISChh1-like transposons (55), mediate the transfer of a variety of genes enhancing the pathogenicity of the host, such as resistance genes and virulence genes (56). In this case, for *C. hyointestinalis* subsp. *hyointestinalis*, the transposon disrupts the *pycB* gene, potentially pseudogenizing it (57). When visualizing the subgraph of the transposon, with all paths traversing ≥90% of the LMEM nodes, we found this transposon in twenty different genomes. While this information offers valuable insights in its abundance, it also underscores the challenges of large-scale analyses. For example, the use of less stringent aligners can lead to the generation of extensive collections of matches, making it more difficult to select the most similar link. LMEMs help address this challenge by selecting both maximum coverage and identity. As *C. hyointestinalis subsp. hyointestinalis* is an emerging pathogen (37) identifying MGEs and their genomic links with Graphite can help in understanding its genomic evolution and potentially inform surveillance strategies.

While the genetic exchange between different *Campylobacter* species warrants further investigation, the power of Graphite to rapidly detect such events in this example dataset showcases its flexibility in identifying DNA transfer events. The examples above include previously undetected transfers between genomes and chromosomes, showing that LMEM analysis is a treasure trove for evolutionary genomics analysis, and underscoring the value of fast and memory-efficient algorithms to enable exploration of the vast microbial sequence space.

## Supplementary Material

lqae142_Supplemental_Files

## Data Availability

The genome identifiers used in this study are recorded in Supplementary Table S1. The code to run Graphite on Linux is available at https://github.com/MGXlab/Graphite (10.5281/zenodo.13177409) and https://github.com/MGXlab/subgraphs (10.5281/zenodo.13177469).
